# Elevated Levels of Urinary 8-Hydroxy-2′-deoxyguanosine, Lymphocytic Micronuclei, and Serum Glutathione *S*-Transferase in Workers Exposed to Coke Oven Emissions

**DOI:** 10.1289/ehp.8562

**Published:** 2005-12-15

**Authors:** Ai-Lin Liu, Wen-Qing Lu, Zeng-Zhen Wang, Wei-Hong Chen, Wen-Hong Lu, Jing Yuan, Pei-Hong Nan, Jian-Ya Sun, Ya-Lin Zou, Li-Hong Zhou, Chi Zhang, Tang-Chun Wu

**Affiliations:** 1 Department of Occupational and Environmental Health and Ministry of Education Key Lab for Environment and Health, School of Public Health, Tongji Medical College, Huazhong University of Science and Technology, Wuhan, People’s Republic of China; 2 Center for Disease Control and Prevention, Taiyuan Steel and Iron Limited Company, Taiyuan, People’s Republic of China

**Keywords:** coke oven emissions, glutathione *S*-transferase, 8-hydroxy-2′-deoxyguanosine, 1-hydroxypyrene, micronuclei

## Abstract

To investigate associations among occupational exposure to coke oven emissions (COEs), oxidative stress, cytogenotoxic effects, change in the metabolizing enzyme glutathione *S*-transferase (GST), and internal levels of polycyclic aromatic hydrocarbons (PAHs) in coke oven workers, we recruited 47 male coke oven workers and 31 male control subjects from a coke oven plant in northern China. We measured the levels of 1-hydroxypyrene (1-OHP) and 8-hydroxy-2′-deoxyguanosine (8-OHdG) in urine, micronucleated binucleated cells (BNMNs) in peripheral blood lymphocyte, and GST in serum. Our results showed that the group exposed to COEs had significantly increased levels of 1-OHP [median 5.7; interquartile range (IQR), 1.4–12.0 μmol/mol creatinine] compared with the control group (3; 0.5–6.4 μmol/mol creatinine). In addition, the median levels (IQR) of 8-OHdG, BNMNs, and GST were markedly increased in the exposed [1.9 (1.4–15.4) μmol/mol creatinine; 6 (2–8) per thousand; 22.1 (14.9–31.2) U/L, respectively] compared with controls [1.3 (1.0–4.0) μmol/mol creatinine, 2 (0–4) per thousand; and 13.1 (9.5–16.7) U/L, respectively]. These results appeared to be modified by smoking. However, multivariate logistic regression analysis revealed that exposure to COEs had the highest odds ratio among variables analyzed and that smoking was not a significant confounder of the levels of studied biomarkers. Overall, the present findings suggest that COE exposure led to increased internal PAH burden, genetic damage, oxidative stress, and GST activity. The consequences of the changes in these biomarkers, such as risk of cancer, warrant further investigations.

Coke oven emissions (COEs) are formed and released into the environment when coal is pyrolyzed into coke (National Toxicology Program 2002). Epidemiologic studies have shown that occupational exposure to COEs during the coking process lead to increased incidence of pulmonary and prostate cancers among coke oven workers (Costantino et al. 1995). COEs are complex mixtures containing a large number of polycyclic aromatic hydrocarbons (PAHs), which are carcinogenic and mutagenic to humans [International Agency for Research on Cancer (IARC) 1987]. Hence, identification of early biomarkers for occupational exposure to PAHs may lead to effective preventive measures to reduce exposure to COEs and related health effects.

Oxygen radicals generated by environmental agents and endogenous processes may induce damage to DNA (Halliwell 1994). For instance, 8-hydroxy-2′-deoxyguanosine (8-OHdG) represents an important product from oxidative damage to DNA. 8-OHdG is formed in a promutagenic DNA lesion induced by the reaction of hydroxyl radicals with guanosine at the C8 site in DNA (Kasai et al. 1986). Oxidative stress may be implicated in aging, carcinogenesis, and other degenerative diseases, and the analysis of urinary excretion of 8-OHdG is a useful approach to assess individual cancer risk due to oxidative stress (Kasai 1997).

The micronucleus (MN) assay is a widely used genotoxic assay to detect both clastogenic and aneugenic potencies of genotoxic agents or radiation (Ramalho et al. 1988). Numerous epidemiologic studies have suggested that chromosomal alterations including formation of MNs may serve as an effective biomarker to estimate cancer risk (Hagmar et al. 1998). In particular, the MN assay in peripheral blood lymphocytes has been extensively used as a standard method to evaluate the presence and the extent of chromosome damage in workers occupationally exposed to genotoxic agents (Bonassi et al. 2001).

Xenobiotic metabolizing enzymes play a key role in chemical carcinogenesis and are often used as biomarkers to evaluate exposure and effect of organic pollutants (van der Oost et al. 1996). Certain PAHs are bifunctional inducers, which are mediated by aryl hydrocarbon receptor function and enhance the activities of glutathione *S*-transferase (GST) (Gujaeva et al. 1999; Prochaska and Talalay 1988). GST catalyzes the conjugation of electrophilic molecules with glutathione to protect macromolecules from damage (Awasthi et al. 1994). In addition, GST activity has been extensively studied and used as an effective biomarker to monitor water PAH load in the tissue of aquatic organisms (Cairrao et al. 2004; Cheung et al. 2001; Tuvikene et al. 1999). However, no data are available for the effect of occupational PAH exposure on the levels of serum GST activities of coke oven workers.

In the present study, we investigated urinary 8-OHdG and MNs of lymphocytes from coke oven workers who were exposed to COEs. In addition, the activity of serum GST, a universal detoxifying enzyme present in the human body, was examined in these coke oven workers to explore possible modulation on GST activity by PAH exposure. Urinary 1-hydroxypyrene (1-OHP), a recognized biomarker of exposure to PAHs (primary constituents of COEs), was also investigated to estimate the internal burden of PAHs in this study population (Jongeneelen 2001).

## Materials and Methods

### Study populations and sample collection.

The study subjects were the coke oven workers in a steel plant in Taiyuan, northern China, where the workers are exposed to PAHs during the open-air coking process in their daily shift. Seventy-eight male workers selected from this coke oven plant were classified into two groups, COE-exposed and control, based on historic exposure data from mandatory regular air sample analysis. The exposed group consisted of 47 coke oven workers; 31 control subjects were workers of the distillery, maintenance sections, and offices in the same plant. The workers participating in the study had been employed for at least 10 years and were currently working in the coke oven plant. After providing their written informed consent to participate in this study, at enrollment all the individuals were interviewed; a questionnaire was used to elicit their health status, body weight and height, occupational history, smoking, and alcohol consumption.

The one-time blood (2 mL each) and urine samples (20 mL each) were collected from subjects at the end of the work shift. Fresh blood lymphocyte cultures were carried out for the MN analysis. Serum was separated by centrifugation and stored at −80°C for detection of GST activity. The urine samples were stored at −28°C before being analyzed for 1-OHP and 8-OHdG. The research protocol was approved by the Ethics and Human Subject Committee of Tongji Medical College.

### Measurement of urinary 1-OHP.

We determined urinary 1-OHP using high-performance liquid chromatography (HPLC) according to Li et al. (2003). We used 2 mL urine from each sample for the detection assay, in which 0.5 mL of sodium hydroxide (15 mol/L) was added, and the samples were incubated at 100°C for 3 hr in the dark. Then, 50 μL carbazole (100 μg/mL) was added to each sample as the internal standard. The samples were adjusted to pH 3–5 with hydrochloric acid (1 mol/L). Subsequently, 1-OHP was extracted from urine with 4 mL dichloromethane. The extracts were evaporated to dryness under vacuum before being dissolved in 0.3 mL HPLC solvent and analyzed by HPLC (Varian, Walnut Creek, CA, USA). The separation of 1-OHP was achieved using a reverse-phase C18 column (Spherisorb ODS2, 4.6 mm × 250 mm, 5 μm; Waters, Milford, MA, USA). The mobile phase used for isocratic elution of 1-OHP was composed of methanol and water (3:1). To quantify 1-OHP, we used a pump system (Varian-Prostar model 230; Varian, Walnut Creek, CA, USA), an autosampler (Varian-Prostar model 410), and a fluorescence detector (excitation wavelength 346 nm, emission wavelength 386 nm; Varian-Prostar model 360). Identification and quantification of 1-OHP were based on retention time and peak area measured using a linear regression curve obtained from internal standard solutions. The detection limit of urinary 1-OHP was 0.5 ng/mL; for measurements below 0.5 ng/mL, we used 0.35 ng/mL (70% of the detection limit) as the default. Urine 1-OHP concentrations were expressed as micromoles per mole creatinine.

### Measurement of urinary 8-OHdG.

The cleanup treatment of urine and analysis of urinary 8-OHdG were carried out according to a method described by Germadnik et al. (1997) with minor modifications. Briefly, urine samples were first centrifuged at 1,500 × *g* for 5 min to remove precipitates. Then, 2 mL supernatant was adsorbed twice on preconditioned cartridges (10 mL/500 mg; Bond Elut LRC C18 OH; Varian) and eluted twice with 50 mM potassium phosphate monobasic (KH_2_PO_4_; pH 7.5) containing 15% and 20% methanol, respectively. Subsequently, the eluate was evaporated to remove methanol, supplemented by HPLC solvent to bring a final volume of 1.5 mL, and analyzed by an HPLC system with electrochemical detector (Varian-Prostar model 370; Varian) equipped with a glassy-carbon working electrode operated at +0.8 V versus a silver/silver chloride reference electrode. The separation of 8-OHdG was carried out on a reverse-phase C18 column with 30°C column temperature. The mobile phase used for isocratic elution of 8-OHdG was composed of 50 mM KH_2_PO_4_ (pH 3.5), 1% methanol, 2.5% acetonitrile, 2 mM KCl, and 0.1 mM EDTA. The flow rate was 0.8 mL/min, and the limit of detection of urinary 8-OHdG was 1 ng/mL. We quantified 8-OHdG in urine by the peak area of measurement using the linear regression curve for standard solutions of 3.5, 14, 56, and 224 nmol/L. For measurements below 1 ng/mL, we used 0.5 ng/mL, half of the detection limit, as the default. The concentration of urinary 8-OHdG is presented as micromoles per mole creatinine.

### Analysis of lymphocytic MNs.

We used a cytokinesis-block MN assay to measure lymphocytic MNs. Fresh blood lymphocyte cultures were set up by adding 0.5 mL whole blood to 4.5 mL RPMI-1640 medium supplemented with fetal calf serum (15%) and penicillin (100 IU/mL). Phytohemagglutinin (Sigma, St. Louis, MO, USA) was added to lymphocyte cultures at a final concentration of 20 μg/mL. After 44 hr of incubation, cytochalasin-B (Sigma) was added to the culture medium at a final concentration of 6 μg/mL to arrest cytokinesis. After a total incubation period of 72 hr, cells were harvested by centrifugation at 400 × *g* for 10 min and mild hypotonic treatment in 0.075 M KCl for 2–3 min at room temperature. The cell suspensions were again centrifuged at 400 × *g* for 10 min. The pellets were fixed twice in freshly prepared cold methanol/acetic acid (5:1) and dropped onto slides before staining with 10% Giemsa solution for approximately 10 min. For each sample, 1,000 binucleated cells and MNs in binucleated cells were examined, and the frequencies of BNMNs were assessed according to the criteria of Kirsch-Volders et al. (2000). One reader blinded to the status of the subjects scored all slides.

### Determination of serum GST activity.

We measured GST activity in serum, calculated as units per liter, using a GST colorimetric activity assay kit (Jiancheng Bio Company, Nanjing, China).

### Statistical methods.

We performed the statistical analyses using SPSS software (version 12.0) for Windows (SPSS, Chicago, IL, USA). We examined the normal distribution of all data using the Shapiro-Wilk normality test to determine subsequent use of appropriate tests for statistical comparison. We used Mann-Whitney and Pearson chi-square tests to compare the demographics and lifestyle variables between the exposure and control groups. The mean values of 1-OHP, 8-OHdG, BNMNs, and GST were calculated after categorizing by smoking status (smokers and nonsmokers), and the data were reported as median and interquartile range because variables were not normally distributed. We used the Mann-Whitney test to compare values of biomarkers between the exposure and control groups. Spearman’s rank correlation coefficient was calculated to evaluate the relations between 1-OHP levels, GST activity, 8-OHdG concentration, and BNMN frequency. We performed multivariate logistic regression to calculate odds ratios (ORs) and 95% confidence intervals (CIs) to assess the impact of independent variables [occupational exposure, body mass index (BMI), smoking, alcohol drinking, and age] on dependent variables (8-OHdG, BNMNs, and GST). For all the tests, *p* < 0.05 was defined as significant with a two-sided test.

## Results

### Demographic characteristics of study subjects.

Table 1 shows the characteristics of study subjects by work site. Coke oven workers were 1 year older (mean age) than control subjects. We found no significant differences in employment time, BMI, or percentages of smokers and alcohol drinkers between the two groups.

### Concentrations of urinary 1-OHP and 8-OHdG, lymphocyte BNMN frequencies, and serum GST activities.

As shown in Table 2, we found a significant difference in urinary 1-OHP between the exposure group and the control group (*p* = 0.033). As an internal exposure biomarker, body PAH burden was associated with coke oven exposure. Likewise, we observed that compared with the control group, coke oven workers had a significant increase of biologic effect biomarkers: 8-OHdG (*p* = 0.022), lymphocyte BNMN formation (*p* = 0.014), and serum GST activity (*p* < 0.001). Furthermore, the results appeared to be modified by smoking. For instance, statistically significant differences between the exposure group and the control group existed only in smokers for 1-OHP (*p* = 0.029), BNMNs (*p* = 0.002), and GST (*p* < 0.001) and in nonsmokers for 8-OHdG (*p* = 0.005). For both exposed and control groups, no statistical differences in these biomarkers were observed between smokers and nonsmokers. However, further multivariate analysis with adjustment for smoking was performed.

### Correlation between 1-OHP, 8-OHdG, BNMNs, and GST.

**Figure 1:**
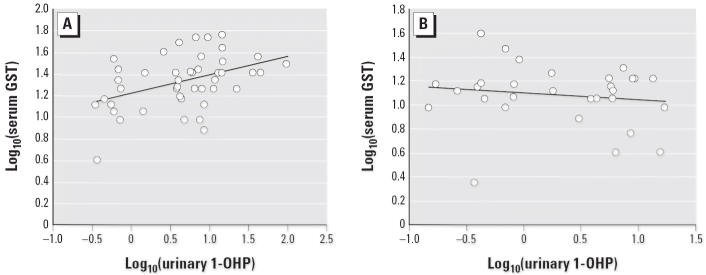
Scatter plot of correlation between log_10_-transformed serum GST activities and log_10_-transformed urinary 1-OHP levels in the exposed group (*A; y* = 0.1709*x* + 1.2198; Spearman rank correlation coefficient = 0.434; *p* = 0.002) and in the control group (*B; y* = −0.0569*x* + 1.1001; Spearman rank correlation coefficient = 0.076; *p* = 0.686).

We observed a significant correlation in urinary 1-OHP and serum GST activities in the total population (exposed and controls) (*p* = 0.005) (Table 3) and in the exposed group (*p* = 0.002) (Figure 1A) but not in the control group (*p* = 0.686) (Figure 1B). However, we found no correlation between 1-OHP and other biomarkers, as shown in Table 3. Additionally, Table 3 presents a significant correlation between lymphocyte BNMN frequencies and serum GST activities (*p* = 0.009).

### Effect of associated variables on 8-OHdG level, GST activity, and BNMN frequency.

Multivariate logistic regression analyses were performed with adjustment for age and smoking to evaluate the possible determinants of increased 8-OHdG levels, BNMN frequency, and GST activity. We calculated OR values of occupational exposure, BMI, smoking, alcohol drinking, and age to compare their contribution with the measured biomarkers. As shown in Table 4, for all three biomarkers that were significantly different between exposure and control groups, the OR values of coke oven exposure were significantly associated with 8-OHdG (OR = 4.4; 95% CI, 1.4–13.7), GST (OR = 13.2; 95% CI, 3.9–44.3), and BNMNs (OR = 2.7; 95% CI, 1.0–7.4), although the association with the BNMN frequency was marginal (*p* = 0.047). Age was associated with an increase in the risk of BNMN formation (OR = 2.7; 95% CI, 1.0–7.3), but BMI was associated with a decrease in the risk of 8-OHdG adduct (OR = 0.3; 95% CI, 0.1–0.8).

## Discussion

Coke oven workers have a high probability of exposure to PAHs during the coking process. Benzo[*a*]pyrene (B[*a*]P) is commonly detected in PAH-containing mixtures. The concentration of B[*a*]P in the air on the top side of the coke oven (0.33 μg/m^3^)—a parameter of external exposure level—was almost 10 times the level in the office (0.04 μg/m^3^) (not shown). Leng et al. (2004b) demonstrated that B[*a*]P exposure in coke oven workers is much higher than that of the control group. The distinct B[*a*]P levels between coke oven workers and control subjects from offices warrants further investigation on internal exposure markers and susceptibility biomarkers. The assessment of internal exposure of coke oven workers to PAHs was based on the determination of urinary 1-OHP (Jongeneelen 2001; Leng et al. 2004a, 2004b). Because PAHs are normally present in cigarettes (Kim et al. 2003), elevated levels of 1-OHP have been confounded by smoking among workers. Bouchard and Viau (1999) reported a synergistic effect on excretion of 1-OHP between smoking and occupational PAH exposure. Indeed, in the present study we also showed a significant increase in urinary 1-OHP in smokers but not in nonsmokers among coke oven workers compared with control subjects. However, the significant smoking-adjusted associations suggest that the internal exposure level of PAHs was due to external exposure, including both smoking and coke oven exposure, in the workers.

Urinary excretion of 8-OHdG, as the repair product from oxidative DNA modification by excision enzymes, is an *in vivo* measure of overall oxidative DNA damage (Cooke et al. 2000, 2005) and has often been used as a biomarker to assess the extent of oxidative DNA damage and repair in the occupational setting (Lagorio et al. 1994; Pilger et al. 2000; Tagesson et al. 1993; Toraason et al. 2001). Although urinary 8-OHdG levels may be affected by renal impairment (Akagi et al. 2003), our participants had no reported history of kidney diseases, which was further confirmed by the normal results of their routine urine analysis at the time of the study. In the present study, the level of urinary 8-OHdG was much higher in coke oven workers than in control subjects. Although elevated 8-OHdG levels existed only in nonsmokers and not in smokers of the exposure group, further multivariate logistic regression analysis suggests that occupational exposure to COEs was associated with an increase but BMI was associated with a decrease in the levels of 8-OHdG after adjustment for age and smoking. BMI showed a significant effect on 8-OHdG excretions, which may be due to higher rate of metabolism in lean subjects with increased availability of reactive oxygen species (Loft et al. 1992).

It is important to investigate the effect of occupational exposure to COEs on genotoxicity in coke oven workers. There was a significant increased frequency of BNMNs in smokers but not in nonsmokers among coke oven workers, when compared with control subjects. The multivariate logistic regression analysis revealed that both occupational exposure to COEs and age were risk factors of formation of MNs in the workers we studied. The same levels of association of occupational exposure and age with the MN formation suggest that they were equally important risk factors. The present finding implies that age as a time-dependent variable should be carefully considered and controlled in similar epidemiologic studies of environmental exposure using MNs as a biomarker.

As an important detoxification enzyme, GST plays a major role in protecting individuals from PAH-induced mutagenesis and carcinogenesis (Nakajima et al. 1995). In the present study, serum GST activity in coke oven workers was much higher than that of controls. Because GST serves as a phase II enzyme in detoxifying xenobiotics and keeping intracellular redox balance in the presence of its substrates, the increase of GST activity in coke oven workers may represent adaptation or defense against the burden of mutagens and carcinogens in the human body. Occupational exposure is a significant risk factor revealed by multivariate logistic regression analysis. However, the other two factors we studied, aging and smoking, were not significant. Furthermore, we found a strong positive correlation between GST activity and PAH exposure based on urinary 1-OHP levels. Additionally, correlation analysis also revealed a significant positive correlation between GST and BNMNs, indicating coherence of toxicity response to PAH exposure. It is widely recognized that GST polymorphisms potentially contribute to individual susceptibility to carcinogen-induced biomarkers of exposure and effect (Wormhoudt et al. 1999). Indeed, Leng et al. (2004b) demonstrated that the frequency of MNs was lower in coke oven workers with a nonnull glutathione *S*-transferase mu-1 (*GSTM1*) genotype than in those with the null *GSTM1* genotype in a steel company in northeastern China. Hence, influence on MN formation by GST polymorphism should be taken into account in future studies. Taken together, GST should be considered a possible indicator for monitoring PAH exposure. To the best of our knowledge, this is the first attempt to estimate the relationship between individual serum GST activity and occupational exposure to PAHs.

As for the correlations among 1-OHP, 8-OHdG, BNMNs, and GST, the 1-OHP concentrations were not significantly correlated with 8-OHdG levels or BNMN frequency in all subjects, suggesting that these markers may have different specificities. It is well known that in, addition to PAHs, COEs also contain a variety of nitrosamines, coal tar, metals compounds, and so forth. These cocontaminants derived from COEs may also contribute to the levels of urinary 8-OHdG and lymphocytic MNs (Kim et al. 2004; Palus et al. 2003).

In summary, we simultaneously investigated levels of PAH exposure, cytogenetic damage, oxidative stress, and enzyme activity change induced in workers exposed to COEs. Our data suggest that urinary 1-OHP and serum GST might be regarded as biomarkers of PAH exposure and that urinary 8-OHdG and lymphocytic MNs could be used as parameters to reflect occupational health effects in coke oven workers. However, confounding factors, such as BMI and age, may influence the assessment of biologic effects of occupational COE exposure. Overall, the present study provides some new clues in developing biomarkers in biomonitoring and early prevention of health effects associated with occupational exposure to COEs, including genotoxicity burden and subsequent cancer risk.

## Figures and Tables

**Table 1:** Characteristics of workers in the exposed and control groups.

Variable	Exposed (*n* = 47)	Controls (*n* = 31)	*p*-Value^a^
Age, year (mean ± SD)	39.9 ± 1.5	38.7 ± 2.4	0.036
Years of employment (mean ± SD)	18.9 ± 4.5	16.8 ± 6.5	0.089
BMI, kg/m^2^ [*n* (%)]			
≤24	18 (38.3)	12 (38.7)	0.971
> 24	29 (61.7)	19 (61.3)	
Smoking [*n* (%)]			
Yes	12 (25.5)	6 (19.4)	0.526
No	35 (74.5)	25 (80.6)	
Alcohol drinking [*n* (%)]			
Yes	27 (57.5)	19 (61.3)	0.786
No	20 (42.5)	12 (38.7)	

a Mann-Whitney test and Pearson chi-square test for comparisons between the exposed and control groups.

**Table 2:** Median levels (IQRs) of biomarkers of subjects in the exposed and control groups.

	Exposed			Controls		
Biomarker	Smokers	Nonsmokers	All	Smokers	Nonsmokers	All
1-OHP	6.8 (2.6–14.5)^a^*	3.0 (0.6–6.9)	5.7 (1.4–12.0)^b^*	3.0 (0.7–7.4)	2.4 (0.4–5.8)	3.0 (0.5–6.4)
8-OhdG	1.9 (1.1–5.5)	2.9 (1.5–30.1)^c^**	1.9 (1.4–15.4)^b^*	1.7 (1.0–4.6)	1.0 (0.7–1.2)	1.3 (1.0–4.0)
BNMNs (per 1,000)	6 (2–8)^a^**	6 (2–7)	6 (2–8)^b^*	2 (0–6)	3 (2–4)	2 (0–4)
GST	25.7 (18.5–34.8)^a^#	16.7 (12.2–25.7)	22.1 (14.9–31.2)^b^#	13.1 (9.5–16.7)	12.9 (11.3–14.9)	13.1 (9.5–16.7)

a Exposed smokers increased compared with control smokers.

b Exposed group increased compared with control group.

c Exposed nonsmokers increased compared with control nonsmokers.

* *p* < 0.05,

** *p* < 0.01, and

# *p* < 0.001 by Mann-Whitney test.

**Table 3:** Correlations of the studied variables among subjects in the exposed and control groups.

	GST		8-OHdG		BNMNs	
Variable	*r*^a^	*p*-Value^b^	*r*^a^	*p*-Value^b^	*r*^a^	*p*-Value^b^
1-OHP	0.314	0.005	0.161	0.160	0.120	0.290
GST			0.108	0.349	0.296	0.009
8-OhdG					0.136	0.235

a Spearman rank correlation coefficient.

b Spearman rank correlation coefficient for the comparisons between each of the studied variables.

**Table 4:** Multivariate logistic regression analysis of risk factors for 8-OHdG, BNMNs, and GST.

	8-OhdG (μmol/mol creatinine)^a^		BNMNs (per thousand)^b^		GST (U/L)^c^	
Variable	*p*-Value	OR (95% CI)	*p*-Value	OR (95% CI)	*p*-Value	OR (95% CI)
COE exposure^d^	0.010	4.4 (1.4–13.7)	0.047	2.7 (1.0–7.4)	< 0.001	13.2 (3.9–44.3)
Age^e^	0.200	0.8 (0.6–1.1)	0.047	2.7 (1.0–7.3)	0.672	1.1 (0.8–1.4)
Smoking^f^	0.196	2.2 (0.7–7.4)	0.550	1.4 (0.4–4.6)	0.141	2.6 (0.7–9.2)
BMI (kg/m^2^)^g^	0.018	0.3 (0.1–0.8)	—	—	—	—
Alcohol drinking^h^	0.066	0.4 (0.1–1.1)	—	—	—	—

a 8-OHdG: > 1.8 (1), ≤ 1.8 (0) (1.8: median level of all subjects).

b BNMNs per 1,000 binucleated cells: > 4 (1), ≤ 4 (0) (4: median level of all subjects).

c GST: > 16.7 (1), ≤ 16.7 (0) (16.7: median level of all subjects).

d COE exposure: subjects with COEs (1), subjects without COEs (0).

e Age: > 40 (1), ≤ 40 (0) (40: median level of all subjects).

f Smoking: yes (1), no (0).

g BMI: > 24 (1), ≤ 24 (0).

h Alcohol drinking: yes (1), no (0).
